# An Exceptional Clinical Presentation associating an occipital stroke, a Superior and Inferior Mesenteric Thrombosis following Covid 19 disease, case report

**DOI:** 10.1016/j.amsu.2021.103082

**Published:** 2021-12-01

**Authors:** Siham Elmir, Rachid Jabi, Mohammed Noumairi, Mohammed Gartit, Mehdi El bekkaoui, Imane Skiker, Brahim Housni, Mohammed Bouziane, Ahmed Amine El Oumri

**Affiliations:** a Department of Physical Medicine and Rehabilitation, Faculty of Medicine and Pharmacy, Immunohematology/Cellular Therapy Laboratory Adults and Children(LIHTC), University Mohammed First Oujda, Morocco; b Department of General Surgery, Mohammed VI University Hospital, Faculty of Medicine and Pharmacy, Laboratory of Anatomy, Microsurgery and Surgery Experimental and Medical Simulation (LAMCESM),Mohammed Ist University, Oujda, Morocco; c Department of Radiolgy, Mohammed VI University Hospital, Faculty of Medicine and Pharmacy, Laboratory of Anatomy, Microsurgery and Surgery Experimental and Medical Simulation (LAMCESM),Mohammed Ist University, Oujda, Morocco; d Department of Anaesthesia and Intensive Care, Mohammed VI University Hospital, Faculty of Medicine and Pharmacy, Laboratory of Anatomy, Microsurgery and Surgery Experimental and Medical Simulation (LAMCESM),Mohammed Ist University, Oujda, Morocco

**Keywords:** Covid 19, Stroke, Mesenteric thrombosis, Rehabilitation, Anticoagulation

## Abstract

**Introduction:**

The Covid 19 pandemia since the first reported case in 2019 had a direct socioeconomic impact related to morbi-mortality and indirect in response to protection and isolation strategies. To our knowledge thrombo-embolic complications can be a mode of revelation complicating the management.

**Case presentation:**

We present an exceptional case of a patient with a history of Covid 19,admitted 21 days later for disturbed consciousness, in whom an ischemic occipital stroke ,intestinal and colonic ishemia had been objectified. Our objective through this presentation is to remind the thrombo-embolic particularity of Covid 19, to take the viral attack as a serious antecedent in the periods following theinfection and to put the point on the primordial place of early rehabilitation in patients with stroke.

**Discussion and Conclusion:**

We discuss through this report the recommendations of anticoagulation in Covid 19 patients and the place of early rehabilitation in patients with stroke. We also report a new case among the rare cases described in the literature that associates several thrombo-embolic manifestations secondary to Covid 19, in particular the neurological and digestive association.

## Highlights

• Exceptional Clinical Presentation associating an occipital Stroke, a Superior and Inferior Mesenteric Thrombosis following Covid 19 Disease.
• We propose take to the viral attack as a serious antecedent.
• This manuscript can add new perspectives to the management practice of this very rare presentation.

## 1 Introduction

Since its declaration in December 2019 [1], deaths secondary to the covid-19 pandemic have exceeded 4.8 million(2).Although the respiratory tropism had dominated the clinical presentation, other manifestations have enriched,complicated the modes of clinical presentation and therapeutic management whose main pillar was the restoration of lung function [3]. This management is subject to international consensus [4] where anticoagulation is included as an essential therapeutic arsenal [5]. This anticoagulation aims to respond to the complex physiopathology of this virus [6], of which several sporadic thromboembolic manifestation were unusual modes of revelation [7].

We report according SCARE guidelines [8] the case of a 28-year-old woman with a history of mild covid 19 infection who was admitted 21 days later with disturbances of consciousness. The clinical examination initially revealed an obnubilated patient whose investigations showed a double thromboembolic location in the occipital lobe and digestive tract.

The particularity of our exceptional case is defined by the rarity of similar cases associating the thromboembolic complication on several organs published in the literature, by the absence of standardization of such a pathology and by and we propose to take into consideration the Covid 19 attack as a serious antecedent in front of the medical affections.

## 2 Case presentation

A 28 years old patient native and resident in the Moroccan oriental, having as antecedent an infection Covid 19 during the first wave with light respiratory manifestations; of good evolution under symptomatic treatment(Figues1). Three weeks later, the patient consulted the emergency room for consciousness disorder, visual and coordination disorders in an apyretic context in whom the clinical examination found an obnubilated patient, hemodynamically and respiratorily stable with a capillary gylcemie level of 1g/l. She was quickly conditioned and a standard infectious biological workup was performed with a hemogram in favor of anemia with Hb at 9g/dl, a lumbar puncture and bacteriological examination of the urine were normal, and a cerebral imaging showed an occipital stroke with free supra-aortic trunks (Fig. 1a, Fig. 1b, Fig. 1c, Fig. 1d). A multidisciplinary discussion was quickly made opting for a hospitalization in intensive care unit and the realization of an etiological assessment of the young patient.

During her stay in the intensive care unit, the patient had received curative anticoagulation associated with early rehabilitation consisting on prevention of decubitus complications, preservation of functional capital and protection of the environment with rigorous psychological support because our young patient had not initially accepted her ishemic stroke. The thrombophilia investigations performed in favor of thrombocytosis, an increase in the values of D-Dimer, Fibrinogen with a negative *trans*-thoracic ultrasound. A new multidisciplinary discussion was held where post Covid 19 thromboembolic complications were evoked as etiology.On the third day of her admission our patient presented an abdominal distension with disturbance of the infectious balance. An abdominal CT scan was performed in favor of entero-mesenteric ischemia and left colonic ishemia motivating the surgical exploration (Fig. 1a, Fig. 1b, Fig. 1c, Fig. 1d). This exploration was carried out under general anaesthesia by the head of surgery and revealed intestinal necrosis and necrosis of the left colon, which led to an intestinal resection-anastomosis and double-stomy resection of the colon with wide drainage (Fig. 2a, Fig. 2b).

**Fig. 1a:**
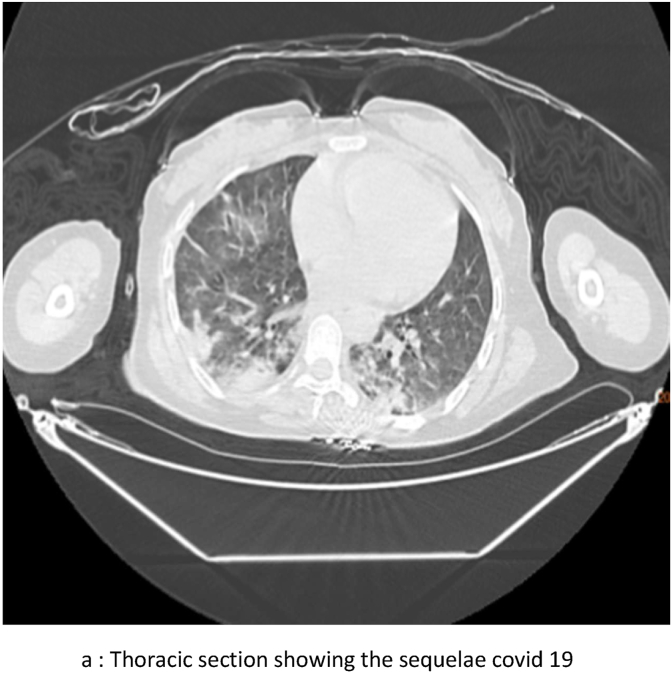
Thoracic section showing the sequelae covid 19.

**Fig. 1b:**
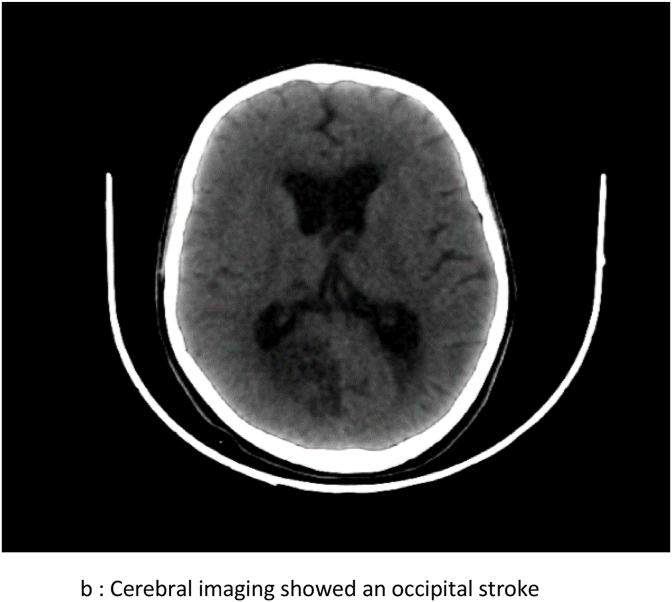
Cerebral imaging showed an occipital stroke.

**Fig. 1c:**
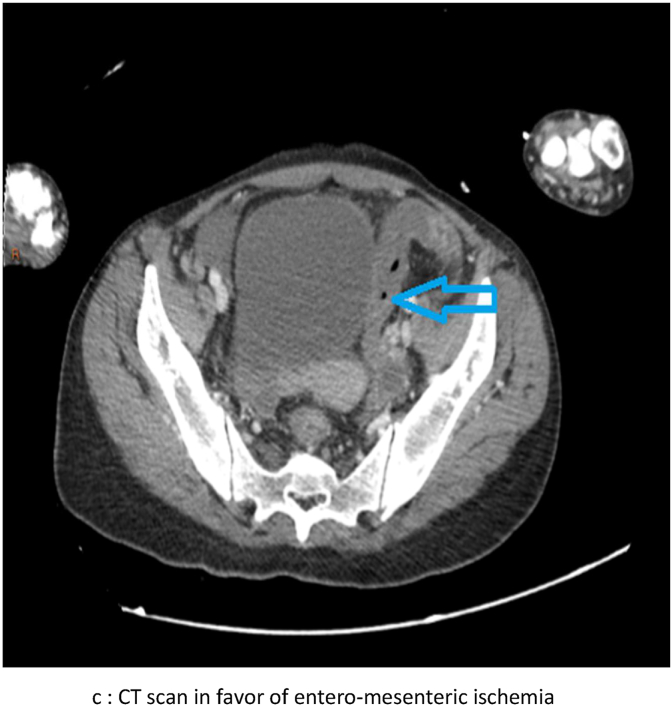
CT scan in favor of entero-mesenteric ischemia.

**Fig. 1d:**
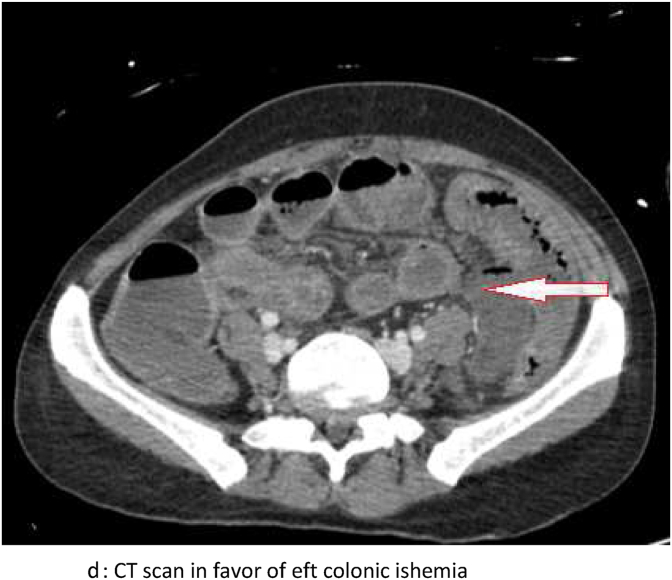
CT scan in favor of eft colonic ishemia.

**Fig. 2a:**
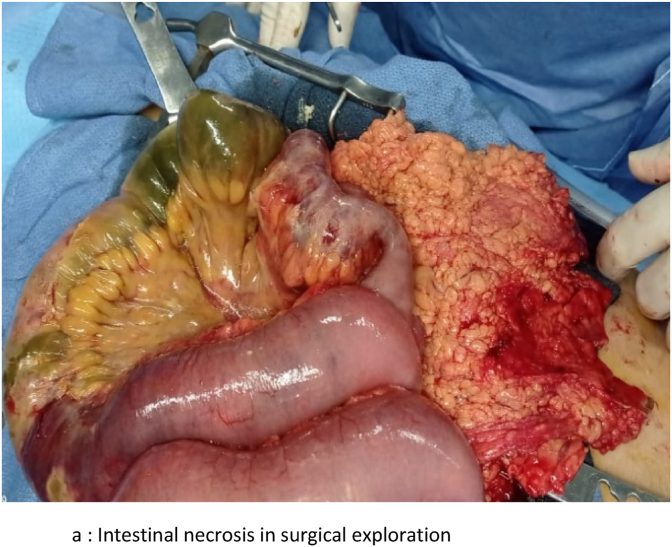
Intestinal necrosis in surgical exploration.

**Fig. 2b:**
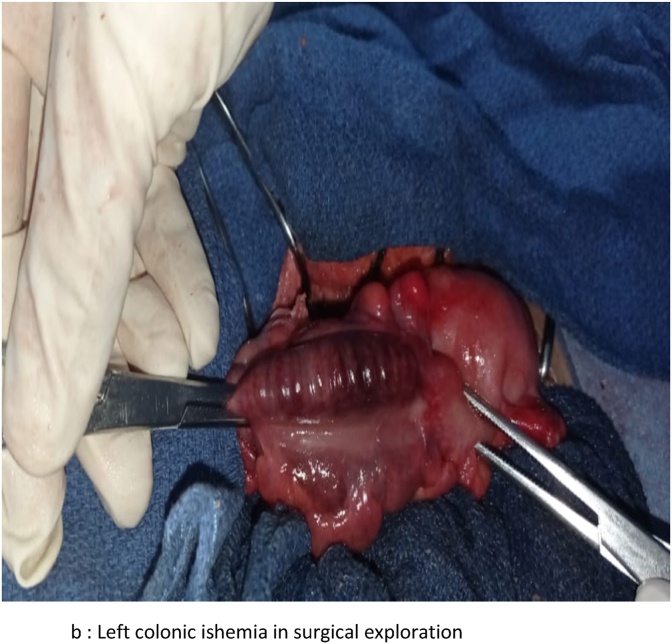
Left colonic ishemia in surgical exploration.

The postoperative evolution went well with gradual return of autonomy, the discharge from the hospital was done at 10 days of hospitalization. A rigorous follow-up by a multidisciplinary team consisting of psychological support, active rehabilitation and care of the colonic stoma. Three months later, a restoration of continuity was carried out in our patient who was satisfied with the care overall despite the difficult and unpleasant management of the stoma.

## 3 Discussion

Since the declaration of the first case of Covid 19 virus in Wuhan, China in December 2019 [1], the number of direct deaths related to the virus has exceeded 4.8 million cases until early October 2021 [2].

Certainly that respiratory involvement dominated the clinical picture and constituted the mainstay of management against this new fatal Virus [3],but the extra-respiratory manifestations often complicated the management [9].

This is explained by the complex pathophysiology of this virus [6] whose disruption of coagulation mechanisms has been the subject of several researches [10] and the prescription of preventive anticoagulation came out as a recommendation of several scientific committees such as American Society of Hematology (ASH) [5]. In spite of the advent of diagnostic means; in particular Next-generation sequencing (NGS) based which is a powerful technique but limited by the enormous variation in viral genome [11]; the asymptomatic forms pose a problem for the control of this epidemic by increasing the risk of contamination [12] and no health structure has proposed a systematic screening of asymptomatic persons given the reliable profitability and the important cost of this action [13].

Among the extra-respiratory manifestations explained by coagulation disturbance, some authors report stroke as a complication or mode of revelation [14] whose treatment is essentially based on revascularization followed by secondary prevention [15] and on the unavoidable role of rehabilitation [16,17].

This rehabilitation is subject to international guidelines [18] which give the best results by starting the rehabilitation procedure as early as possible [19].

Similarly, following the pathophysiological logic, some reports describe Acute intestinal ischemia as a revealing or complicating mode of management [20], which may or may not be associated with other gastrointestinal manifestations that several authors have discovered by performing abdominal imaging in Covid patients [21].

The particularity of our case is summarized in the association of two cerebral, then intestinal thromboembolic complications in a young patient with a history of mild Covid symptomatology [4], and in whom classical etiological exploration was negative.

This presentation, which associates thrombosis in two different stages,was described in the form of rare and isolated case reports in the literature. Azouz E et al. reported an entero-mesenteric infarction in a 56 year old patient, an occlusion of the middle cerebral artery, and a thrombosis of the aortic arch [22].Also, the involvement of both superior and inferior mesenteric pedicles in Covid 19 patients as in our patient was rarely reported and is only published as sporadic cases [7]. As our patient had initially benefited from medical treatment according to the consensus [4] and in whom preventive anticoagulation had been indicated according to the meta-analysis published by McBane RD 2nd [23], the neurological and then digestive manifestations can be explained by the severity of the systemic inflammation and viral endotheliitis and the installation of the coagulopathy [24]. It should also be noted that although anticoagulation improves the risk of thromboembolism in Covid 19 patients [25], it does not guarantee protection against this fatal pathology [26]. On the other side the place of rehabilitation and mobilization is essential during all the steps of the management [27] and the vaccination against this virus guarantees the primary prevention with less new severe incident cases [28].

We propose as a take home message to take the covid 19 infection as a serious medical history in front of the clinical manifestations following this attack and highlighting the importance of anticoagulation as an effective therapeutic weapon and the precocity of a rehabilitation in patients with deficiencies.

## Patient perceptive

The procedure of surgery was explained to the patient with all advantages and possible complications. He agreed on the procedure and informed consent was taken from her.

## Ethics approval

Not applicable.

## Funding

The author(s) received no financial support for the research, authorship and/or publication of this article.

## Author's contribution

Siham Elmir: Writing, review and editing of the manuscript.

Jabi Rachid, Houda Mirali, Mohammed Noumairi, Mohammed Gartit: Contributed for diagnose and treatment of the patient.

Mohammed Bouziane, Brahim Housni and El Oumri Ahmed Amine: Review, Supervision and surgeons of the patient.

Registration of research studies: Our paper is a case report; no registration was done for it.

Guarantor: Siham Elmir.

## Consent of patient

Written informed consent was obtained from the patient for publication of this case report and accompanying images. A copy of the written consent is available for review by the Editor-in-Chief of this journal on request.

## Registration of research studies

Not applicable.

## Guarantor

Dr. Siham Elmir.

## Provenance and peer review

Not commissioned, externally peer reviewed.

## Sources of funding

We don't have any financial sources for our research.

## Declaration of competing interest

All authors disclose any conflicts of interest.
